# Junipers of Various Origins as Potential Sources of the Anticancer Drug Precursor Podophyllotoxin

**DOI:** 10.3390/molecules26175179

**Published:** 2021-08-26

**Authors:** Diana I. Ivanova, Paraskev T. Nedialkov, Alexander N. Tashev, Marta Olech, Renata Nowak, Yana E. Ilieva, Zlatina K. Kokanova-Nedialkova, Teodora N. Atanasova, George Angelov, Hristo M. Najdenski

**Affiliations:** 1 Institute of Chemical Engineering, Bulgarian Academy of Sciences, 1113 Sofia, Bulgaria; georgeangelov@yahoo.com; 2 Faculty of Pharmacy, Medical University, 1000 Sofia, Bulgaria; pnedialkov@gmail.com (P.T.N.); illievayana@gmail.com (Y.E.I.); zlatina.kokanova@gmail.com (Z.K.K.-N.); thedi_7@abv.bg (T.N.A.); 3 Department of Dendrology, University of Forestry, 1756 Sofia, Bulgaria; altashev@abv.bg; 4 Department of Pharmaceutical Botany, Faculty of Pharmacy, Medical University of Lublin, 20-059 Lublin, Poland; marta.olech@umlub.pl; 5 The Stephan Angeloff Institute of Microbiology, Bulgarian Academy of Sciences, 1113 Sofia, Bulgaria; hnajdenski@abv.bg

**Keywords:** cytotoxic agents, *Juniperus* L., podophyllotoxin, LC-MS, HRMS

## Abstract

Juniper representatives are natural sources of plenty of bioactive metabolites and have been used since ancient times as folk remedies against tapeworms, warts, cancer, etc. The antiproliferative activities of junipers are attributed to podophyllotoxin (PPT), which is a precursor for the synthesis of efficient anticancer drugs. However, the natural sources of PPT, *Sinopodophyllum hexandrum* (Royle) T. S. Ying and *Podophyllum peltatum* L., are already endangered species because of their intensive industrial exploitation. Therefore, identification of other sources of PPT is necessary. This study is a broad comparative investigation of junipers, for which original sources have been accessed from different continents of the world. The present research is aimed at the identification of species, producing PPT and other lignans at concentrations that are sufficient for the high antiproliferative activity of the corresponding extracts. Cytotoxic juniper leaf extracts demonstrated a broad spectrum of activity on a panel of cancer cell lines. The antiproliferative properties of junipers were attributed to the combined activity of great diversity of lignans (podophyllotoxin, deoxypodophyllotoxin, β-peltatin, yatein, matairesinol, anhydropodorhizol, etc.), detected by UHPLC-HRMS and LC-ESI-MS/MS in the corresponding extracts. Several species of the genus *Juniperus* L. were outlined as perspective sources of drug precursors with potential pharmaceutical applications.

## 1. Introduction

The genus *Juniperus* L. (Cupressaceae) comprises about 50–75 species, depending on the taxonomic classification [1], as well as more than 220 cultivars [2]. Junipers belong to the Pinophyta division (Coniferae) of plants, producing plenty of bioactive metabolites, contributing to their various biological activities [3]. Since ancient times junipers have been known as folk remedies against various diseases. The first information about the healing properties of junipers has been found in the Egyptian Papyrus Ebers (c. 1500 B.C.), which informed about the application of junipers against tapeworms and roundworms [4]. The prominent review of Hartwell (1967–1971) on medicinal plants reported the application of *Juniperus virginiana* L. (red cedar) leaves for the treatment of venereal warts [5]. The usage of the red cedar against warts and tapeworms was also confirmed by other authors [6]. The work of Hartwell was extended by Graham et al. [7], who informed about the prevention and treatment of cancer by *Juniperus recurva* Buch.-Ham. ex D. Don and *Juniperus squamata* Buch.-Ham. ex D. Don in the traditional medicine of Nepal [8]. Various antibacterial, antifungal, and antiviral activities of *J. communis* have been also reported [9,10]. Juniper essential oils (*J. communis*, *J. virginiana*, *J. excelsa*, *J. oxycedrus,* etc.) have been used to treat wounds, acne, skin infections [11], etc. Abortifacient effects of junipers (*J. communis*, *J. virginiana,* etc.) have also been reported [12,13].

**Figure 1:**
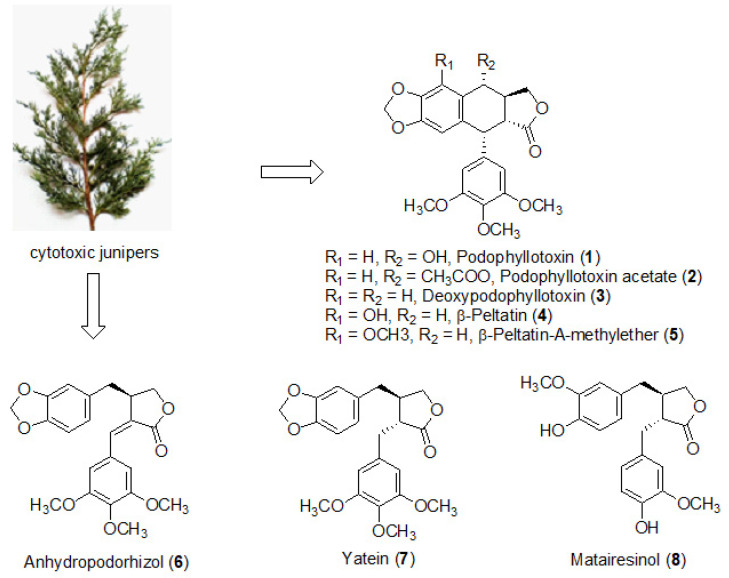
Structures of the lignans **1**–**8**, identified in the cytotoxic juniper leaf extracts.

Podophyllotoxin (PPT, Figure 1) is a secondary metabolite that mediates the antiviral, anthelmintic, anticancer, and other activities of junipers. PPT was isolated in the 1880s from the American mayapple (*Podophyllum peltatum* L.) and its structure was elucidated in the 1930s [14]. Both Indian physicians and Western physicians in New Orleans have used mayapple for the treatment of genital warts [15]. At present, PPT is used in the topical agent Podofilox (containing 0.5% PPT) against *Condyloma acuminatum* (genital warts), caused by the human papillomavirus.

Due to its various biological activities, PPT attracted scientific attention as a lead compound for the design of different drugs. Structural modifications of the original PPT molecule (glycosylation, acetalization, etc.) were aimed at increasing the water solubility and decreasing of the toxicity of the corresponding drugs. After screenings of hundreds of substances over a period of 20 years, the PPT derivatives Etoposide, Etopophos, and Teniposide were discovered. Today, these drugs belong to the golden standards in the therapy of lung, breast, ovarian, testicular, stomach, bladder, pancreatic, brain, blood cancers [16,17,18] etc. The combination of PPT with cisplatin has been useful in the treatment of neuroblastoma and the combination of PPT derivatives with interferon has shown efficacy against genital infections [19]. PPT analogs have been applied to treat other infections, such as malaria, psoriasis, etc. [20]. Moreover, recent clinical trials develop Etoposide as drug to treat cytokine activation in COVID-19 patients, caused by SARS-CoV-2 (“Etoposide in patients with COVID-19 infection”, clinicaltrials.gov, NTC 04356690).

At present, natural sources of PPT are *Sinopodophyllum hexandrum* (Royle) T. S. Ying (Himalayan mayapple) and several species of the genus *Podophyllum* L. (Berberidaceae), e.g., *Podophyllum peltatum* L. (American mayapple). The resin, obtained from the roots and rhizomes of *P. peltatum,* was found to contain about 10% PPT, whereas the resin from the Indian *S. hexandrum* was reported to contain more (about 40%) PPT [21]. However, the PPT content in the Himalayan species, estimated by the weight of dry roots and rhizomes, is highly variable (0.01–5.5%), which reveals a difficult reproducibility of the PPT delivery from the roots and rhizomes of these plants [22,23]. That is why the leaves of *Podophyllum* species were studied as alternative sources of PPT. The American mayapple leaves were found to contain 0.6–3% PPT [24], but later studies revealed that some representatives of the species contained up to 4.5% PPT in their leaves [25]. The PPT concentration in the Indian mayapple leaves was reported also to be widely distributed in the range of 1–6%, based on the dry raw material weight [26]. The PPT biosynthesis in these species is highly variable, depending on the plant age, growing conditions, such as altitude and shade/sun exposure [22,24]. In addition, the American and Himalayan mayapples have become an endangered species because of their intensive industrial exploitation. 

Therefore, other natural sources of PPT are necessary for the pharmaceutical industry. Plenty of plants were screened for various cytotoxic activities, based on their content of PPT and other lignans. Plant species belonging to more than 60 families, including genera other than the genus *Podophyllum,* were found to produce PPT and derivatives: *Jeffersonia, Diphylleia* and *Dysosma* (Berberidaceae); *Catharanthus* (Apocynaceae); *Polygala* (Polygalaceae); *Anthriscus* (Apiaceae); *Linum* (Linaceae); *Hyptis*, *Teucrium*, *Nepeta* and *Thymus* (Lamiaceae); *Thuja, Juniperus*, *Callitris* and *Thujopsis* (Cupressaceae); *Cassia* (Fabaceae), *Haplophyllum* (Rutaceae); *Commiphora* (Burseraceae); *Hernandia* (Hernandiaceae), etc. [27]. However, difficulties in cultivation hampered the extraction of PPT from plants. In addition, only some of the species, including junipers, were found to contain PPT or its derivatives at concentrations, ensuring efficient activity of the corresponding extracts.

Junipers are natural factories for the biosynthesis of nearly 580 compounds, including cytotoxic lignans (PPT and other derivatives), sesquiterpenes, diterpenes, flavonoids, etc., responsible for their diverse biological activities [28]. Recently, the content of various polyphenols and antioxidant activity of about 30 junipers of different origins was reported [29]. Junipers are evergreen plants that have the advantage to produce huge amounts of plant mass all throughout the year. In addition, studies of a juniper representative (*J. virginiana*) found no significant interindividual and seasonal changes in its biosynthesis of PPT, being independent from the collection site [30]. Therefore, the genus *Juniperus* attracted an increased scientific attention for finding of plant species as potential sources of podophyllotoxin and other drug precursors.

PPT, deoxy-PPT, and/or their derivatives, considered as potential anticancer agents [31], were found in various juniper species: *J. virginiana* L. [32,33,34], *J. × media* Pfitzeriana [35], *J. bermudiana* L. [30], *J. horizontalis* Moench [36], *J. communis* L. [37], *J. sabina* L. [38,39,40,41], juniper cultivars [42], etc. Identification of other lignans was reported only for a few juniper species: yatein in *J. chinensis* L. [43]; matairesinol in *J. martinezii* Pérez de la Rosa; yatein and matairesinol in *J. squamata* Buch.-Ham. ex D. Don and *J. virginiana* L. [44]; anhydropodorhizol and β-peltatin derivatives in *J. sabina* [38]; β-peltatin-A-methyl ether in *J. phoenicea* L. [45]. The presence of other lignans in addition to PPT derivatives and/or deoxy-PPT set the pattern for efficient antiproliferative activity of the corresponding juniper extracts. Indeed, β-peltatin-A-methyl ether [46], yatein [47], and anhydropodorhizol [48] were reported to inhibit efficiently various cancer cells proliferation. A synergistic action of matairesinol with the leading anticancer drug doxorubicin has been observed, suggesting a potential use of this compound in adjuvant anticancer therapy [49,50]. 

To our knowledge, the present research is the first broad comparative investigation of the *Juniperus* species, with original sources being accessed from different continents around the world. The study is aimed at the identification of PPT and other lignans in junipers and the evaluation of the antiproliferative activity of the corresponding juniper extracts. Ultra-high performance liquid chromatography, coupled to high-resolution mass spectrometry (UHPLC-HRMS), and LC-ESI-MS/MS-MRM were used for the analysis of the cytotoxic metabolites in the studied junipers. As a result, in the cytotoxic juniper extracts, a great diversity of lignans was identified. Their combined activity set the pattern for efficient cytotoxic properties of the corresponding leaf extracts. Several lignans, such as β-peltatin derivatives, yatein, matairesinol, and anhydropodorhizol were identified in addition to PPT and/or deoxy-PPT for the first time in many cytotoxic juniper extracts. Several species of the genus *Juniperus* of various origins were outlined as potential natural sources of drug precursors with expected pharmacological applications.

## 2. Results

### 2.1. Quantitative Determination of the Podophyllotoxin Content in the Juniper Extracts

The identification of plant species as sources of efficient cytotoxic agents is a permanent challenge in the anticancer research. In this connection, junipers, originating from various continents of the world, were assayed for the quantitative determination of the PPT content and antiproliferative activity evaluation of their extracts. The accession summaries of the original juniper sources are presented in Table 1.

Podophyllotoxin was identified using LC-ESI-MS/MS by comparing its retention time and MRM (Multiple Reaction Monitoring) transitions with the values and transitions from a standard compound tested under the same conditions. The calibration curve for the quantification of PPT was obtained in MRM mode for the transition 415 *m*/*z* → 397 *m*/*z* (Table 2). Quantification was based on the corresponding peak area. The limits of detection (LOD) and quantification (LOQ) for PPT (Table 3) were determined at a signal-to-noise ratio of 3:1 and 10:1, respectively, by injecting a series of dilute solutions with known concentrations.

### 2.2. Identification of PPT Derivatives and Other Lignans in the Cytotoxic Juniper Extracts

**Figure 2:**
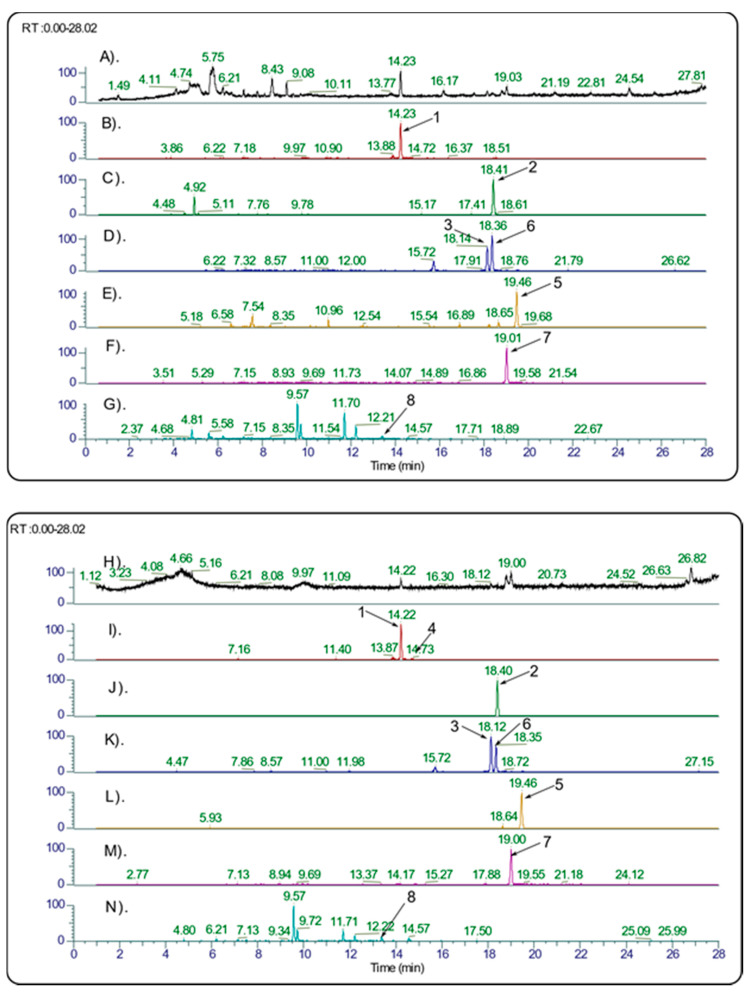
Example UHPLC chromatograms of cytotoxic juniper leaf extracts. The lignan numbers, shown on the chromatograms with arrows, correspond to Figure 1. (**A**) Total Ion Chromatogram (TIC) of Grey Owl juniper extract (extract № 7, Table 1); (**B**–**G**) Extracted Ion Chromatograms (XIC) of Grey Owl juniper extract at *m*/*z* 415.1387, 457.1496, 399.1438, 429.1544, 401.1595, and 359.1489, respectively, with a 5 ppm isolation window; (**H**). TIC chromatogram of *J. virginiana* L. extract (extract №1, Table 1); (**I**–**N**) XIC chromatograms of *J. virginiana* L. extract at *m*/*z* 415.1387, 457.1496, 399.1438, 429.1544, 401.1595, and 359.1489, respectively, with a 5 ppm isolation window.

The identification of various lignans in the juniper extracts was carried out by UHPLC coupled to HRMS (Figure 2). Generally, the leaf extracts contained more PPT than the galbuli extracts (Table 1). Thus, the leaf extracts were subjected to further detailed investigation. High-resolution MS/MS fragmentation analysis of the juniper leaf extracts identified a great diversity of lignans, for which structures were assigned in correspondence with the literature data (Figure 1, Table 4).

### 2.3. Identification of Juniperus Species with Efficient Antiproliferative Activity of Their Extracts

In this research, NB-4 acute promyelocytic leukemia (APL) cells were used as a model cancer cell line to study the antiproliferative activity of various juniper extracts. This cell line is characterized by a balanced reciprocal chromosomal translocation *t*(15;17), involving fusion of the retinoic acid receptor alfa (RARα) gene to the promyelocytic leukemia (PML) gene, leading to formation of an oncogene PML-RARα with abnormal properties [53]. This oncogene prevents the normal maturation of the promyelocytes to granulocytes, inducing accumulation of immature white blood cells in APL patients. All-*trans*-retinoic acid (ATRA) and anthracycline antibiotics induce remission in up to 80–90% of the PML-RARα positive patients. However, about one-quarter of the patients become resistant or suffer from life-threatening retinoic acid syndrome (characterized by fever, dyspnea, vomiting, etc.). Thus, new cytotoxic agents are required for the therapy of APL and other types of cancer. In continuation of previous studies [40,42], where juniper representatives were found to possess superior antiproliferative activity, the genus *Juniperus* attracted our attention for the identification of species with efficient biosynthesis of cytotoxic PPT and other lignans with perspectives to be used as anticancer drug precursors. Plenty of junipers, for which original sources were accessed from different continents of the world, were studied in this research for their antiproliferative activity.

The antiproliferative activity of the studied plant extracts was evaluated by their half-maximum growth-inhibitory concentrations (IC_50_), calculated from the corresponding dose–response curves of MTT-assay of treated NB-4 cells. Lower IC_50_ values indicated higher activity of the extracts.

Representatives of several juniper species with high antiproliferative activity of their leaf extracts in NB-4 cells were also assayed on a panel of other cancer cell lines (Table 5). These extracts demonstrated highly efficient activity in K-562 human chronic myeloid leukemia (CML) and BV-173 human B cell precursor leukemia (Philadelphia chromosome-positive cell lines, bearing *t*(9;22) BCR-ABL1 fusion gene); T-24 human urinary bladder carcinoma and HT-29 human colon adenocarcinoma cell lines.

## 3. Discussion

### 3.1. Quantification of PPT and Identification of Other Lignans in the Juniper Leaf Extracts

The concentration of podophyllotoxin in the studied extracts was determined by comparison with a standard compound (Table 1). In addition, the UHPLC, coupled to high-resolution MS/MS fragmentation analysis, referred to the literature data, revealed that the cytotoxic juniper species produce a diversity of other lignans in addition to PPT (Table 4) [54,55,56]. Matairesinol and yatein were supposed to be biosynthetic precursors of PPT [16], β-peltatin, and deoxypodophyllotoxin [57]. Anhydropodorhizol was also identified as a secondary metabolite. Thus, plenty of other lignans in addition to previously detected PPT, were found in the studied juniper leaf extracts of *J. virginiana*, *J. sabina*, *J. scopulorum*, *J. horizontalis*, *J.* *× media*, and *J. chinensis* representatives (Table 4), of which the combined activity contributed to high antiproliferative properties of the corresponding extracts.

The extracts of *J. pinchotii*, *J. ashei*, *J. deltoides*, and *J. excelsa* representatives with the lowest cytotoxic activity in NB-4 cells (Table 1) contained negligible amounts of PPT and other lignans, or these compounds were not found.

### 3.2. Selection of Juniper Species with High Antiproliferative Activity, Based on the PPT Content of Their Extracts

The antiproliferative properties of the studied junipers are a result of the combined action of a diversity of lignans in addition to PPT in the cytotoxic leaf extracts. The highest PPT content and antiproliferative activity, respectively, were obtained for the leaf extracts of *J. virginiana* L. (incl. its cultivars ‘Glauca’, ‘Cinerascens’, ‘Grey Owl’), *J. sabina* L., (incl. *J. sabina* var. *balkanensis*), *J. scopulorum* ‘Moon light’, *J. chinensis* ‘Pfitzer Mattews Blue’, *J. chinensis* ‘Plumosa Aurea’, *J. horizontalis* Moench, and various *J. × media* hybrids. The PPT concentrations of these leaf extracts varied in the range of 0.2–1.3% PPT and their IC_50_ values were found in the range of 0.2–0.7 μg/mL (Table 1).

The biosynthesis of cytotoxic metabolites is determined by the plant genome, as only distinct species of the genus *Juniperus* possess cytotoxic properties. For example, *J. × media* hybrids, studied here, must have inherited efficient biosynthesis of cytotoxic metabolites from their parent species *J. sabina* and *J. chinensis* [58]. ‘Grey Owl’ juniper is considered to be a hybrid of *J. virginiana* and *J. × pfitzeriana* (synonym *J. × media*). Thus, the genome of three species was supposed to be involved in the activity of the ‘Grey Owl’ cultivar, because *J. × pfitzeriana* is a hybrid of *J. chinensis* and *J. sabina*.

A recently identified juniper representative, namely *Juniperus sabina* var. *balkanensis* R. P. Adams and A. N. Tashev, also demonstrated superior antiproliferative properties in the group of studied junipers. *J. sabina* var. *balkanensis* grows naturally in the Balkan Peninsula (Albania, Bosnia-Herzegovina, Bulgaria, Croatia, Macedonia, Greece, Turkey, etc.) and Italian regions [59,60]. This juniper variety has inherited the cytotoxic properties of its predecessors by hybridization of *Juniperus sabina* and *Juniperus thurifera*, when the natural habitats of these species overlapped in ancient times [61].

Considering the potential use of junipers as industrial crops for the delivery of drug precursors, the shrub-like representatives were considered as more perspective plants for cultivation and industrial applications than the magnificent trees. *J. virginiana* and its cultivars ‘Glauca’, ‘Cinerascens’, as well as *J. scopulorum* ‘Moon light’ grow as magnificent trees. However, junipers that grow as shrubs are easier for cultivation. Hence, shrub-like representatives *J. sabina*, *J. horizontalis*, *J. virginiana* ‘Grey Owl’, various *J. × media* hybrids, *J. chinensis* ‘Plumosa Aurea’, and *J. chinensis* ‘Pfitzer Mattews Blue’, which produce PPT in concentrations, sufficient for high antiproliferative activities of their leaf extracts, were considered as preferable sources of PPT for industrial cultivation.

Other junipers, which leaf extracts also exhibited efficient antiproliferative properties, such as *J. communis* L., *J. chinensis* L., *J. sibirica* Burgsd, *J. pigmaea* K. Koch, *J. formosana* Hayata, etc., were found to contain predominantly deoxypodophyllotoxin and other lignans instead of PPT (Table 4), as was detected by UHPLC/HRMS and MS/MS-fragmentation analysis. The IC_50_ values of these leaf extracts showed also excellent antiproliferative activity on NB-4 cells and varied in the range of 0.5–5 μg/mL (Table 1). Deoxypodophyllotoxin (deoxy-PPT) is a strong cytotoxic agent; however, the absence of a hydroxyl group in its molecule restricts the possibilities for the reduction of its toxicity, because the formation of glycoside derivatives is prevented. This structural specificity of deoxy-PPT limits its perspectives for pharmaceutical applications.

The less efficient activity was determined for the leaf extracts of *J. pinchotii* Sudw., *J. deltoides* R. P. Adams, *J. ashei* J. Buchholz, *J. excelsa* M. Bieb. Their IC_50_ values after treatment of NB-4 cells were determined in the range of 17–137 μg/mL, consistent with the observation that all analyzed lignans persisted in these extracts in negligible amounts or were not detected.

The broad spectrum of the antiproliferative activity of juniper species with a high cytotoxic activity in NB-4 cells was confirmed also by their excellent IC_50_ values after treatment of other cancer cell lines: K-562 human chronic myeloid leukemia and BV-173 human B cell precursor leukemia (Philadelphia chromosome positive cell lines); T-24 human urinary bladder carcinoma and HT-29 human colon adenocarcinoma (Table 5). This result revealed excellent perspectives of the genus *Juniperus* as a potential source of anticancer agents for the treatment of various forms of cancer.

To our knowledge, the present research is the first comparative analysis of the PPT content in the extracts of plenty of species of the genus *Juniperus*, with original sources being accessed from various continents around the world. For the first time, matairesinol, yatein, and anhydropodorhizol were identified in many of the studied juniper extracts in addition to previously detected in some junipers PPT, deoxy-PPT, and β-peltatin derivatives. Their combined activity contributed to the efficient antiproliferative properties of the corresponding cytotoxic leaf extracts.

The juniper representatives with efficient biosynthesis of antiproliferative agents, identified in this research, revealed the genus *Juniperus* around the world as a perspective natural source of drug precursors for the pharmaceutical industry.

## 4. Materials and Methods

### 4.1. Chemicals and Reagents

Podophyllotoxin (standard compound, ≥98%), MTT [3-(4,5-dimethylthiazol-2-yl)-2,5-diphenyltetrazolium bromide], LC grade acetonitrile, and RPMI 1640 medium were purchased from Sigma-Aldrich Fine Chemicals (Saint Louis, MO, USA), Fetal calf serum (FCS) for cell culture was purchased from Biochrom GmbH (Berlin, Germany), and DMSO was purchased from Fluka Chemie AG (Buchs, Switzerland). LC grade water was prepared using a Millipore Direct-Q3 purification system (Bedford, MA, USA).

### 4.2. Plant Material

Junipers from the Arnold Arboretum (AA), Harvard University, Boston, USA, were collected in June 2017 (species № 7–10, 24–26, 32, 35) and in October 2018 (species № 1–6, 12–21, 31). The accession summaries and the original sources of the specimen were reported (Table 1). Junipers from the Balkan Peninsula region were collected as follows: *J. communis* L. was from the village Ognyanovo, Blagoevgrad Province, Rhodope Mountains (41°37′47.3″ N; 23°47′14.5″ E, 700 m a.s.l., April 2017); *J. sibirica* Burgsd. was from the Vitosha mountain, on the outskirts of Sofia (42°34′59.6″ N; 23°17′28.6″ E, 1803 m a.s.l., April 2017); *J. pigmaea* C. Koch was from the Smolyan Province, Mursalitsa region of the Rhodope Mountains (41°38′40.8″ N; 24°29′58.5″ E, 1898 m a.s.l., May 2017); *J. deltoides* R. P. Adams was from the village Ognyanovo, Blagoevgrad Province, Rhodope Mountains (41°37′46.6″ N; 23°47′15.4″ E, 695 m a.s.l., April 2017); *J. excelsa* M. Bieb. was from the reserve Tisata, on the riverside of Struma (41°44′01.6″ N; 23°09′22.5″ E, 199 m a.s.l., April 2017). *Juniperus sabina* var. *balkanensis* R. P. Adams and A. N. Tashev were collected from the eastern Rhodopes (peak Veikata), Bulgaria (November 2017). *J. virginiana* L. (sample № 4) was collected from the Arboretum of the University of Forestry, Sofia, Bulgaria (April 2017). Their voucher specimens were deposited in the Herbarium (SOM) of the Institute of Biodiversity and Ecosystem Research, Bulgarian Academy of Sciences (IBER-BAS). Plant species from the Balkan Peninsula region were authenticated by A. N. Tashev (University of Forestry, Sofia, Bulgaria) and according to R. P. Adams (Adams, 2014).

### 4.3. Extraction Procedure

The plant material was dried for a week at room temperature and then kept in the freezer (−20 °C) in vacuum bags until extraction. For the preparation of the extracts, the plant material (5 g) was ground and mixed with methanol (50 mL, 80% *v*/*v*) in an Erlenmeyer flask with a stopper. The suspension was stirred for 1.5 h in a shaker water bath at 20 °C. The mixture was filtered and the extract was collected. The remaining solid material was subjected to a second extraction for 1.5 h with a new portion of 80% methanol (50 mL). After filtration, the solid mass was stirred again for 1.5 h in 80% methanol (25 mL). The combined extracts were concentrated by a vacuum evaporator. During the vacuum evaporation, a chlorophyll-containing dark green oil appeared in traces amounts (2–4% yield), and was removed by decantation from the main extract. All extracts were freeze-dried (24 h, −50 °C, 0.1 mbar) and kept in the freezer (−20 °C) until analyses.

### 4.4. Cell Culture and MTT-Assays

Cancer cell lines NB-4 acute promyelocytic leukemia, K-562 human chronic myeloid leukemia, BV-173 human B cell precursor leukemia, T-24 human urinary bladder carcinoma, and HT-29 human colon adenocarcinoma were purchased from DSMZ, Germany. The cells were cultured in a humidified incubator (37 °C, 5% CO_2_) in RPMI-1640 with 10% FCS, L-glutamine (2 mM), and HEPES buffer (25 mM). Stock solutions of extracts (20 mg/mL in DMSO) were diluted with the cell culture medium to obtain the desired concentrations. The solvent in the medium was less than 0.5% (*v*/*v*). Cells (3 × 10^5^ cells/mL) were seeded into 96-well plates (100 µL/well) and after 24 h of incubation, the cells were exposed for 72 h to various extract concentrations. MTT assays were performed as described previously [62,63].

### 4.5. Data Processing and Statistics

MTT-assays were carried out in at least four experiments. MTT data were fitted to dose–response curves and IC_50_ values were calculated using non-linear regression analysis by GraphPad Prism (version 6.01 for Windows, GraphPad Software, San Diego, CA USA, www.graphpad.com, accessed on 1 July 2021). Statistical analysis exploited Student’s *t*-test with *p* ≤ 0.05 set as the lowest level of statistical significance.

### 4.6. LC-ESI-MS/MS Analyses

Reversed-phase high-performance liquid chromatography coupled to electrospray ionization mass spectrometry (LC-ESI-MS/MS) was performed on an Agilent 1200 Series HPLC system (Agilent Technologies, Wood Dale, IL, USA) equipped with a binary gradient solvent pump, a degasser, an autosampler, and a column oven connected to 3200 QTRAP Mass spectrometer (AB Sciex, Foster City, CA, USA). Chromatographic separations were carried out on an Eclipse XDB-C18 column (4.6 × 50 mm, 1.8-µm particle size; Agilent Technologies, USA) maintained at 2 5 °C. The gradient program consisted of mobile phase A (0.1% formic acid in water) and mobile phase B (acetonitrile containing 0.1% formic acid) at 0–0.5 min hold at 35% B, a linear ramp to 53% B at 1 min, a hold at 53% B until 4 min, a linear ramp to 90% B at 5 min, and a hold at 90% B until 6 min. The total run time was 9 min, including a 2 min equilibration. The injection volume was 5 μL with a mobile phase flow rate of 450 μL/min.

The QTRAP-MS system was equipped with an electrospray ionization source (ESI) operated in the positive-ion mode, with a capillary temperature of 500 °C and source voltage of 4500 V. Nitrogen was used as a curtain and collision gas. The optimum conditions of the Multiple Reaction Mode (MRM) were determined in the infusion mode. The data were acquired and processed using Analyst 1.5 software (AB Sciex, USA). Triplicate injections were made for each standard solution and sample. Before injection, the samples were subjected to solid-phase purification with Sep-Pak C18 Cartridges (Waters, Dublin, Ireland).

### 4.7. UHPLC-HRMS Analyses

Ultra-high-performance liquid chromatography (UHPLC) coupled to high-resolution mass spectrometry (HRMS) was performed on a Thermo Scientific Dionex Ultimate 3000 RSLC system, connected to a Thermo Scientific Q Exactive Plus mass spectrometer (Bremen, Germany), equipped with Thermo Scientific Xcalibur 3.0 software. AkzoNobel Kromasil Externity XT-1.8-C18 (Bohus, Sweden) narrow-bore column (2.1 × 100 mm, 1.8 µm) with Phenomenex Security Guard ULTRA UHPLC EVO C18 (Torrance, CA, USA) was used and maintained at 40 °C. The mobile phase consisted of systems A (0.1% formic acid in water) and B (0.1% formic acid in acetonitrile). The following gradient was used: the mobile phase was held at 5% B for 0.5 min, gradually turned to 60% B for 22.5 min, kept at 60% B for 2 min, followed by a gradual increase to 85% B for 2.5 min, kept at 85% B for 2 min, and the system was turned to the initial condition of 5% B for 0.5 min. The system was conditioned at 5% B for 4.5 min before injection. The flow rate and injection volumes were 300 µL/min and 2 µL, respectively. Prior to injection, samples were subjected to solid-phase purification by Sep-Pak C_18_ Cartridges (Waters, Ireland), prepared for sample loading using 80% (*v*/*v*) methanol.

## 5. Conclusions

Junipers are evergreen plants, producing plenty of metabolites all the year. The present study is a comparative investigation of the podophyllotoxin concentration, other lignans identification and antiproliferative activity of plant extracts, obtained from a great diversity of junipers, with original sources being accessed from different continents around the world. The chromatography analysis coupled to mass spectrometry showed that many of the studied junipers produce cytotoxic podophyllotoxin at concentrations that are sufficient for the high antiproliferative activities of the corresponding leaf extracts (Table 1).

In general, the juniper leaf extracts contained more PPT than the corresponding galbuli extracts.

The highest concentrations of PPT and best cytotoxic activity of the corresponding leaf extracts, respectively, were determined for *J. virginiana* (including its cultivars ‘Glauca’, ‘Cinerascens’, ‘Grey Owl’), *J. scopulorum* ‘Moon light’, *J. chinensis* ‘Pfitzer Mattews Blue’, *J. chinensis* ‘Plumosa Aurea’, *J. horizontalis*, various *J. × media* hybrids, and *J. sabina*, including *J. sabina* var. *balkanensis*. These extracts contained 0.2–1.3% PPT and their efficient IC_50_ values were found in the range of 0.2–0.7 μg/mL.

Junipers that grow as shrubs are considered to be species with more potential for cultivation and industrial exploitation than representatives that grow as large trees. In this respect, shrub-like representatives *J. sabina*, *J. horizontalis*, various *J. × media* hybrids, *J. virginiana* ‘Grey Owl’, etc. were outlined in this research as preferable sources of PPT for industrial cultivation.

The broad spectrum of activity of highly efficient cytotoxic juniper extracts, detected initially on NB-4 APL cells, was also demonstrated on a panel of other cancer cell lines, including K-562 human chronic myeloid leukemia and BV-173 human B cell precursor leukemia (Philadelphia chromosome positive cell lines), T-24 human urinary bladder carcinoma, and HT-29 human colon adenocarcinoma.

In addition to previously detected PPT and β-peltatin derivatives in some junipers, other lignans, such as matairesinol, yatein, and anhydropodorhizol, were also found for the first time in a number of juniper species (Table 4). The combined activity of the identified lignans set the pattern for the efficient antiproliferative properties of the corresponding juniper leaf extracts.

In conclusion, a number of juniper species of different origins around the world were revealed to be natural factories for the efficient biosynthesis of cytotoxic podophyllotoxin and other lignans that represent the genus *Juniperus* as a source of drug precursors, with potential pharmacological perspectives in the treatment of cancer and other diseases.

## Figures and Tables

**Table 1:** Original source accession summary of junipers of different origins and comparison of the podophyllotoxin content, antiproliferative activity in NB-4 cells, and yields of the juniper extracts.

Extract №	Specimen Number	Juniper Species	F/M	Organ Used	Original Source Accession Summary	%PPT ± SD in the Dry Extract	NB-4 IC_50_ ± SD [μg/mL]	Q%
1	AA 1746–81/A	*J. virginiana* L.	F	leaves	wild, USA, Bald Head Cliff, Maine, Ogunquit, 1981	0.91 ± 0.01	0.21 ± 0.01	12
2	AA 1746–81/A	*J. virginiana* L.	F	galbuli	wild, USA, Bald Head Cliff, Maine, Ogunquit, 1981	0.23 ± 0.01	0.54 ± 0.03	6
3	AA 4714/A	*J. virginiana* ‘Cinerascens’	F	leaves	Berlin, Späth Arb., 1901	1.03 ± 0.03	0.37 ± 0.03	12
4	SOM 174406	*J. virginiana* L.	ME	leaves	cultivar, UFA, Sofia, BG	0.37 ± 0.01	0.48 ± 0.10	15
5	AA 14882/A	*J. virginiana* ‘Glauca’	F	leaves	Rochester Park, New York, 1903	0.74 ± 0.01	0.29 ± 0.02	8
6	AA 14882/A	*J. virginiana* ‘Glauca’	F	galbuli	Rochester Park, New York, 1903	0.04 ± 0.01	0.26 ± 0.01	9
7	AA 1136–61/A	*J. virginiana* ‘Grey Owl’	F	leaves	Dominion Arb., Ottawa, Canada, 1961	0.39 ± 0.01	0.72 ± 0.10	11
8	AA 1136–61/A	*J. virginiana* ‘Grey Owl’	F	galbuli	Dominion Arb., Ottawa, Canada, 1961	0.09 ± 0.01	0.49 ± 0.20	7
9	AA 211–57/A	*J. scopulorum* ‘Moon light’	M	leaves	USA, D. Hill Nurs., Dundee, Illinois, 1957	0.85 ± 0.02	0.20 ± 0.04	18
10	AA 14868/E	*J. sabina* L.	F	leaves	wild, 1904, Uzbekistan	0.50 ± 0.01	0.34 ± 0.01	13
11	SOM 177009	*J. sabina* var. *balkanensis* Adams & Tashev	M	leaves	wild, from eastern Rhodopes, BG	0.20 ± 0.01	0.29 ± 0.03	16
12	AA 1164–56/A	*J. horizontalis* Moench	F	leaves	D. Hill Nurs., USA, Dundee, Illinois, 1956	0.38 ± 0.01	0.23 ± 0.03	12
13	AA 1164–56/A	*J. horizontalis* Moench	F	galbuli	D. Hill Nurs., USA, Dundee, Illinois, 1956	0.03 ± 0.01	2.43 ± 0.22	7
14	AA 74–42/C	*J. × media* ‘Pfitzeriana Argentea’	M	leaves	Morris Arb, Pennsylvania, USA, 1942	0.42 ± 0.01	0.51 ± 0.07	11
15	AA 183–62/A	*J. × media* ‘Óld Gold’	M	leaves	Grootendorst Nurs., Holland, 1962	0.47 ± 0.01	0.42 ± 0.04	10
16	AA 639–48/A	*J. × media* ‘Richeson’	M	leaves	Armstrong Nurs., Ontario, CA, 1948	0.22 ± 0.01	0.43 ± 0.03	13
17	AA 1–51/A	*J. chinensis* ‘Pfitzer Mattews Blue’	M	leaves	Interstate Nursery, Iowa, USA, 1951	1.30 ± 0.05	0.24 ± 0.01	13
18	AA 219–61/A	*J. chinensis* ‘Plumosa Aurea’	M	leaves	Pennsylvania, USA, 1961	0.22 ± 0.02	0.45 ± 0.02	10
19	AA 265–33/A	*J. chinensis* L.	M	leaves	California, USA, 1933	BQL	0.5 ± 0.3	14
20	AA 14809/A	*J. chinensis* L.	F	leaves	Royal Botanic Gardens, UK, Kew, 1908	BQL	1.0 ± 0.1	15
21	AA 14809/A	*J. chinensis* L.	F	galbuli	Royal Botanic Gardens, UK, Kew, 1908	BQL	0.8 ± 0.1	7
22	SOM 174400	*J. communis* L.	F	leaves	wild from Rhodopes, BG	BQL	1.0 ± 0.4	15
23	SOM 174400	*J. communis* L.	F	galbuli	wild from Rhodopes, BG	BQL	3.6 ± 1.7	13
24	AA 49–66/A	*J. communis* ‘Laxa’	M	leaves	U. S. Natl. Arb., 1966, Washington	BQL	0.8 ± 0.2	16
25	AA 4176–1/A	*J. communis* ‘Oblonga Pendula’	M	leaves	USA, Biltmore Estate, North Carolina, 1907	BQL	4.7 ± 0.6	17
26	AA 280–98/A	*J. formosana* Hayata	F	leaves	wild, from Taiwan, 1998	BQL	1.7 ± 0.3	17
27	SOM 174401	*J. sibirica* Burgsd.	F	leaves	wild, from Vitosha, BG	ND	3 ± 1	15
28	SOM 174401	*J. sibirica* Burgsd.	F	galbuli	wild, from Vitosha, BG	BQL	15 ± 3	9
29	SOM 174402	*J. pigmaea* K. Koch	F	leaves	wild from Rhodopes, BG	BQL	5 ± 1	15
30	SOM 174402	*J. pigmaea* K. Koch	F	galbuli	wild from Rhodopes, BG	BQL	29 ± 7	11
31	AA 20–89/A	*J. squamata* ‘Meyeri’	F	leaves	Hicks Nurs., Westbury, NY, 1989	BQL	6.3 ± 0.5	10
32	AA 642–88/B	*J. pinchotii* Sudw.	M	leaves	wild, USA, Oklahoma, Kiowa reserv., 1988	BQL	17 ± 2	19
33	SOM 174403	*J. deltoides* R. P. Adams	F	leaves	wild from Rhodopes, BG	BQL	66 ± 8	16
34	SOM 174403	*J. deltoides* R. P. Adams	F	galbuli	wild from Rhodopes, BG	ND	70 ± 5	17
35	AA 276–86/A	*J. ashei* J. Buchholz	M	leaves	wild, USA, Oklahoma, Murray, 1986	BQL	130 ± 18	28
36	SOM 174404	*J. excelsa* M. Bieb.	F	leaves	wild, Struma riverside, BG	ND	137 ± 12	19
37	SOM 174404	*J. excelsa* M. Bieb.	F	galbuli	wild, Struma riverside, BG	ND	188 ± 55	16
-	control	Podophyllotoxin		-	standard compound	-	0.005 ± 0.001	-

Legend: PPT—podophyllotoxin; NB-4—acute promyelocytic leukemia cell line; AA—specimen of the Arnold Arboretum, Harvard University, Boston, USA, a capital letter qualifier is applied to each individual plant and the year of the original source accession is given; SOM—specimen of the Herbarium of the Institute of Biodiversity and Ecosystem Research, Bulgarian Academy of Sciences; UFA—University of Forestry Arboretum, Sofia, Bulgaria; Arb.—Arboretum; Nurs.—Nursery; F/M—female or male representative; ME—monoecious; NB-4 IC_50_: half-maximum growth-inhibitory concentrations of the extracts in NB-4 cells, given in micrograms of the dry extract per milliliter of cell culture medium. Lower IC_50_ values denote higher activity; Q—yield [%] of the extract related to the weight of the starting plant material; BQL—below the quantification limit (peak detected, concentration > LOD, but < LOQ); LOD/LOQ—limit of detection/quantification; ND—not detected.

**Table 2:** LC-ESI-MS/MS parameters for identification of podophyllotoxin (PPT).

Compound	RT [min]	Molecular Weight	[M + H]^+^ [*m*/*z*]	Fragment Ions [*m*/*z*]	Collision Energy [eV]	DP [V]	EP [V]	CEP [V]	CXP [V]
PPT	3.60	414	415	397	18	30	10	23.6	2
				247	20	30	10	23.6	2
				229	20	30	10	23.6	2

Abbreviations: RT—retention time, given in minutes; [M + H]^+^ protonated molecular ion; *m*/*z*—mass-to-charge ratio; declustering potential (DP); entrance potential (EP); collision cell entrance potential (CEP); collision cell exit potential (CXP).

**Table 3:** Analytical parameters of the LC-MS/MS quantitative method and data for calibration curves for the determination of the podophyllotoxin (PPT) concentration.

Compound	LOD [ng mL^−1^]	LOQ [ng mL^−1^]	*r* ^2^	Equation	Linearity Range [ng mL^−1^]
PPT	5	12.5	0.9992	*y* = 87.3*x* − 144	12.5 to 500

Legend: LOD—limit of detection; LOQ—limit of quantification; *r*—regression coefficient.

**Table 4:** HRMS/MS analysis of lignans, identified in cytotoxic juniper leaf extracts.

Compound	RT min	Molecular Formula	[M + H]^+^			HRMS/MS Fragments **	Extract №	Ref
			Calcd. *	Found	Δppm *			
Podophyllotoxin	14.23	C_22_H_22_O_8_	415.1387	415.1385	−0.61	397.1277 (100), 313.1066 (10), 247.0598 (85), 229.0493 (20), 185.0596 (5)	1, 3–5, 7, 9–12, 14–18 ***	[51,52]
Podophyllotoxin acetate	18.41	C_24_H_24_O_9_	457.1496	457.1493	0.57	397.1287 (80), 355.1169 (70), 313.1070 (100), 229.0492 (50), 185.0596 (25)	7, 9, 10, 14–16, 18	[46]
Deoxypodophyllotoxin	18.14	C_22_H_22_O_7_	399.1438	399.1437	−0.23	231.0649 (100), 187.0752 (10)	1, 3–5, 7, 9–12, 14–20, 22, 24–27, 29	[51,52]
β-Peltatin	14.72	C_22_H_22_O_8_	415.1387	415.1390	0.54	247.0599 (100), 203.0701 (5), 189.0545 (2)	1, 4, 5, 14–20,	[51,52]
β-Peltatin-A-methyl-ether	19.47	C_23_H_24_O_8_	429.1544	429.1546	0.57	261.0755 (100), 217.0860 (10)	1, 3, 4, 7, 9, 10, 12, 14–19, 22, 24, 25	[51,52]
Anhydropodorhizol	18.35	C_22_H_22_O_7_	399.1438	399.1440	0.38	381.1327 (30), 363.1227 (10), 231.0649 (90), 203.0701 (40), 187.0752 (20), 181.0857 (10), 135.0441 (15)	1, 3–5, 7, 9–12, 14–19	[52]
Yatein	19.02	C_22_H_24_O_7_	401.1595	401.1598	0.74	383.1484 (100), 365.1381 (5), 223.0963 (5), 181.0858 (30), 161.0596 (10), 135.0441 (5)	1, 3, 5, 7, 9–12, 14–20, 26	[51,52]
Matairesinol	13.40	C_20_H_22_O_6_	359.1489	359.1490	0.15	341.1379 (60), 323.1274 (25), 223.0964 (10), 163.0752 (10), 137.0596 (100)	1, 3–5, 7, 9, 10, 20, 24, 25, 29	[51]

Legend: RT—retention time in minutes by UHPLC analysis. * The molecular mass of the protonated molecules and Δppm values were determined by *Thermo Scientific Xcalibur* 4.0 software (Waltham, MA, USA). ** Fragmentations are with normalized collision energy (NCE) 10; the ion abundance in brackets is given in %; *** Traces of PPT were found also in the leaf extracts № 19, 20, 22, 24–26, 29, 31–33, and 35. The extract numbers refer to Table 1.

**Table 5:** Antiproliferative activity presented as IC_50_ values [μg/mL] of the corresponding cytotoxic juniper leaf extracts after treatment of a panel of cancer cell lines.

Extract №	*Juniperus* Representatives	K-562	BV-173	T-24	HT-29
4	*J. virginiana*	0.4 ± 0.1	0.14 ± 0.06	1.3 ± 0.5	5 ± 1
7	*J. virginiana* ‘Grey Owl’	0.4 ± 0.3	0.11 ± 0.05	4.0 ± 3.5	8 ± 5
9	*J. scopulorum* ‘Moon light’	0.3 ± 0.1	0.22 ± 0.03	0.8 ± 0.2	2 ± 1
10	*J. sabina*	0.4 ± 0.1	0.24 ± 0.03	0.8 ± 0.3	2 ± 1
11	*J. sabina* var. *balkanensis*	0.2 ± 0.1	0.16 ± 0.03	0.7 ± 0.3	2 ± 1
22	*J. communis*	1.6 ± 1.2	1.6 ± 0.2	19 ± 12	17 ± 7
control	Podophyllotoxin	0.006 ± 0.003	0.007 ± 0.002	0.007 ± 0.002	0.007 ± 0.002

Legend: Cancer cell lines: K-562 human chronic myeloid leukemia (CML), BV-173 human B cell precursor leukemia (Philadelphia chromosome-positive cell lines, bearing *t*(9;22) BCR-ABL1 fusion gene); T-24 human urinary bladder carcinoma; HT-29 human colon adenocarcinoma; IC_50_—half-maximum growth-inhibitory concentration of the extracts in the corresponding cancer cells, given in micrograms of the dry extract per milliliter of the cell culture medium. Lower IC_50_ values denote higher activities.

## Sample Availability

Samples of the compounds are not available from the authors.
